# Dabigatran-induced esophagitis: A case report

**DOI:** 10.1097/MD.0000000000019890

**Published:** 2020-04-24

**Authors:** Yi Zhou, Yancheng Dai, Lei Lu, Zhiquan Fu

**Affiliations:** a Department of Gastroenterology, Shanghai Hospital of Integrated Traditional Chinese and Western Medicine; b Institute of Digestive Diseases, LongHua Hospital; c Department of Pathology, Shanghai Hospital of Integrated Traditional Chinese and Western Medicine, Shanghai University of Traditional Chinese Medicine, Shanghai, China.

**Keywords:** case report, dabigatran, esophagitis, *Helicobacter pylori*

## Abstract

**Rationale:**

Dabigatran is an anticoagulant medication that has been widely used to prevent strokes caused by atrial fibrillation, deep vein thrombosis, and pulmonary embolism. However, the potential adverse effect of dabigatran of gastrointestinal mucosal injury is often neglected, and even induces esophagitis.

**Patient concerns:**

A 77-year-old woman was admitted to the hospital with symptoms of progressive retrosternal pain, upper abdominal discomfort, and dysphagia.

**Diagnosis:**

Esophagogastroduodenoscopy showed longitudinal sloughing mucosal casts in the distal esophagus. Histological examination showed squamous epithelium with neutrophil infiltration, partial epithelial degeneration, and *Helicobacter pylori*. Based on a literature review, medical history, and imaging examination, the patient was diagnosed with dabigatran-induced esophagitis.

**Interventions:**

The patient recovered with standard *H. pylori* eradication therapy and proton pump inhibitor without discontinuing dabigatran.

**Outcomes:**

After 2 weeks, the retrosternal pain and dysphagia were relieved and upper abdominal discomfort was attenuated.

**Lessons:**

Our case highlights the importance of physicians’ awareness of the clinical and endoscopic characteristics of dabigatran-induced esophagitis and the importance of *H. pylori*-associated tests and eradication if necessary for patients with long-term dabigatran treatment.

## 1 Introduction

Dabigatran is an oral anticoagulant that directly inhibits thrombin. It is used as an alternative to warfarin and has similar efficacy for prevention of stroke caused by atrial fibrillation (AF) and prevention and treatment of venous thromboembolism.^[1,2]^ Recently, a few cases of dabigatran-induced esophagitis (DIE) have been reported.^[3–12]^ Here, we report a case of DIE with *Helicobacter pylori* infection in a female patient, who recovered by standard *H. pylori* eradication therapy and proton pump inhibitor (PPI) without discontinuing dabigatran.

## 2 Case presentation

Patient has provided informed consent for publication of the case. The Ethics Committee of Shanghai Hospital of Integrated Traditional Chinese and Western Medicine approved the protocol for this study.

**Figure 1:**
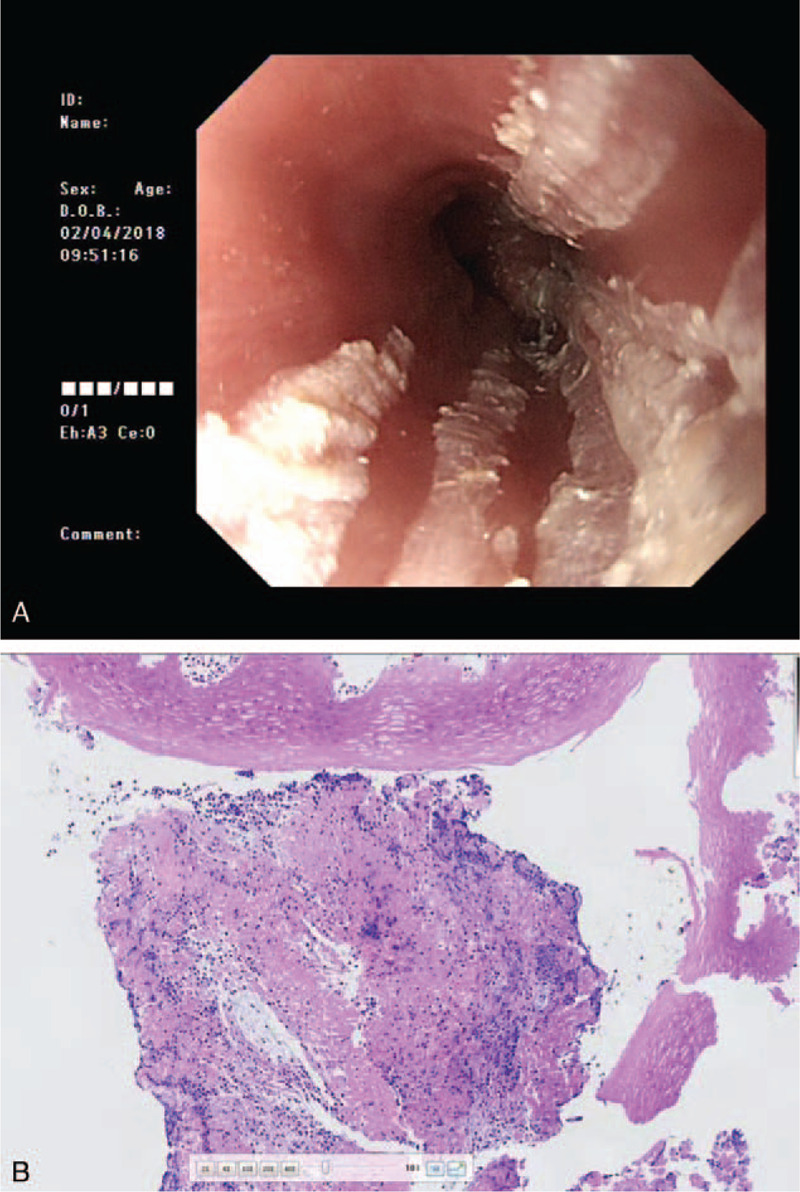
Images on April 2, 2018. A: Esophagogastroduodenoscopy showing longitudinally sloughing mucosal casts in the distal esophagus; B: hematoxylin and eosin staining showing squamous epithelium neutrophil infiltration and partial epithelial degeneration (100×).

A 77-year-old woman presented to the gastroenterology outpatient department with the chief complaints of progressive retrosternal pain, upper abdominal discomfort, and dysphagia which began 1 month ago without obvious causes. The patient had a history of coronary heart disease and AF, and first received radiofrequency ablation in April 2012. The patient had recurrent AF and underwent a second radiofrequency ablation in March 2017. After the second ablation, the patient was on oral dabigatran at 110 mg bid. There was no history of digestive disease in her family. There was tenderness in her mid–upper abdomen. Laboratory results including a complete blood count and electrolytes were in the normal range. Esophagogastroduodenoscopy (EGD) on April 2, 2018, showed mucosal congestion and erosion of the esophagus. Mucosal abscission and multiple necrosis were seen at 20 to 40 cm from the incisor, forming a cast structure with longitudinally sloughing mucosal casts (Fig. 1A). Histological examination of a biopsy specimen showed esophageal squamous epithelium with neutrophil infiltration and partial epithelial degeneration (Fig. 1B). Gastric antrum biopsy demonstrated chronic atrophic gastritis and *H. pylori* (+++).

**Figure 2:**
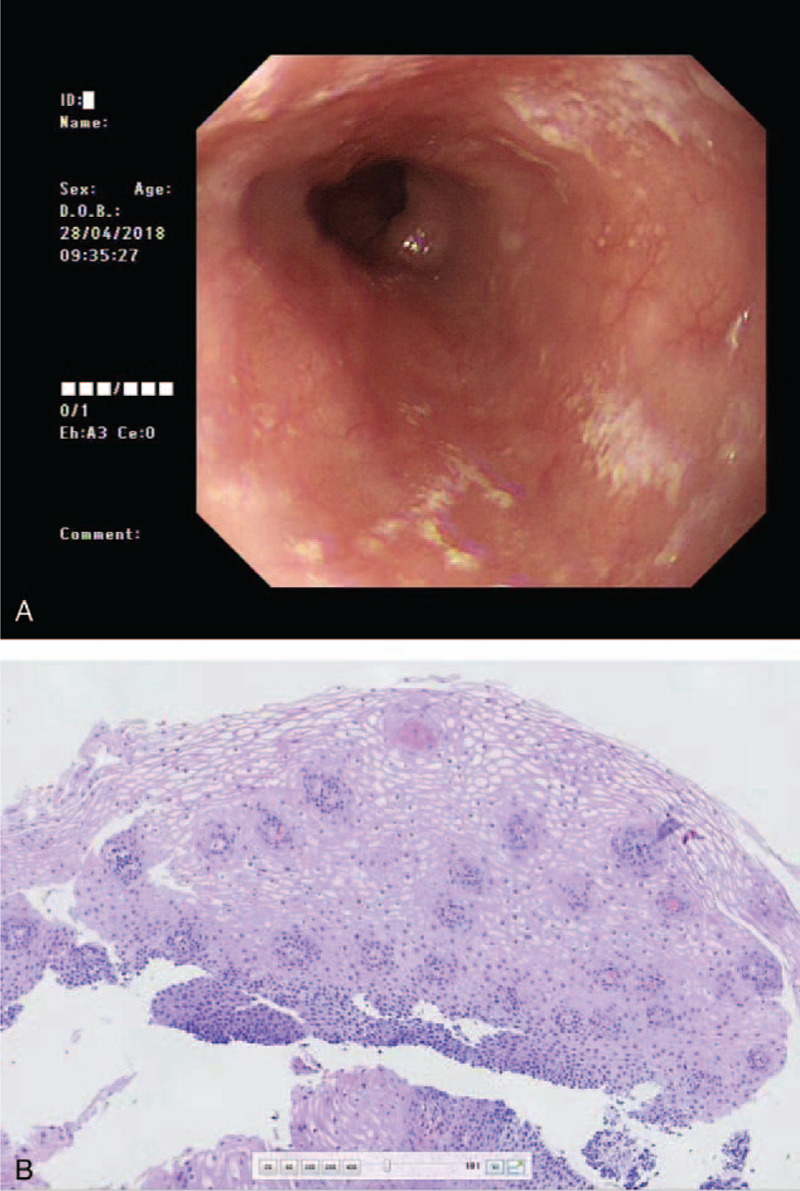
Images on April 28, 2018. A: Esophagogastroduodenoscopy showing scattered erosion in the mid-esophagus; B: hematoxylin and eosin staining showing epithelial hyperplasia (100×).

Based on literature review, medical history, and imaging examination, we speculated that the patient's clinical symptoms were the manifestations of DIE. Initially, the patient was advised to stop taking dabigatran and to start rabeprazole treatment. In the meantime, the patient had AF and had a CHA2DS2-VASC score of 6 points, which is a strong indication for anticoagulant treatment.^[1,2]^ As a result, the patient continued taking dabigatran at the same dose and received standard *H. pylori* eradication therapy, including rabeprazole 20 mg bid, colloidal bismuth pectin 200 mg bid, amoxicillin 1000 mg bid, and clarithromycin 500 mg bid. We also advised her to drink more water after taking dabigatran. After 2 weeks, the retrosternal pain and dysphagia were relieved, along with attenuated upper abdominal discomfort. On April 28, 2018, EGD was performed again. The esophageal mucosa appeared smooth and soft, and scattered erosion was visible at 28 to 32 cm from the incisor (Fig. 2A). Pathological examination showed esophageal squamous epithelium with epithelial hyperplasia (Fig. 2B). Gastric antrum biopsy demonstrated chronic atrophic gastritis and was negative for *H. pylori* (−).

**Figure 3:**
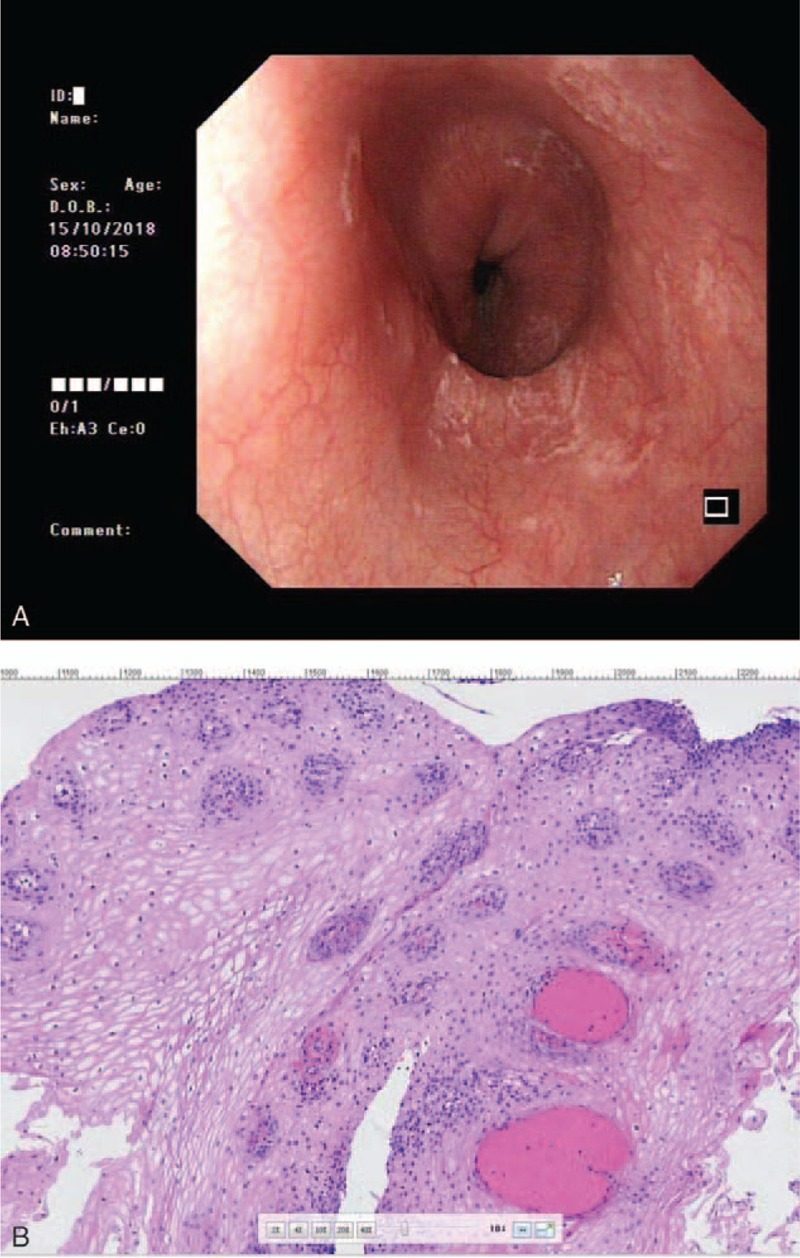
Images on October 15, 2018. A: Esophagogastroduodenoscopy showing scattered white plaques in the distal esophagus; B: hematoxylin and eosin staining showing slight hyperplasia of mucous squamous epithelium, subepithelial vasodilation, hyperemia, interstitial chronic inflammatory cells, leukocyte infiltration, and local lymphoid follicle (100×).

Since the patient still required anticoagulant treatment, she was advised to continue treatment with rabeprazole 20 mg qd for 2 months. The third EGD on October 15, 2018, showed that the esophageal mucosa was smooth and soft, with scattered white plaques in the lower esophagus, and patchy white coating at 32 to 39 cm from the incisor, with a clear dentate line (Fig. 3A). Pathological examination revealed slight hyperplasia of mucous squamous epithelium, subepithelial vasodilation, hyperemia, interstitial chronic inflammatory cells, leukocyte infiltration, and local lymphoid follicles in the esophagus (Fig. 3B). Gastric antrum biopsy demonstrated chronic atrophic gastritis and was negative for *H. pylori* (−).

## 3 Discussion

Dabigatran is a thrombin inhibitor that acts by binding and blocking thrombogenic activity and preventing thrombus formation. It is recommended to reduce the risk of stroke and systemic embolism in patients with nonvalvular AF as the level of effort B by the American Heart Association.^[2]^ However, dabigatran capsules contain tartaric acid, which lowers gastric pH and needs to be fully absorbed. The lower pH is associated with dyspepsia. Some researchers assume that it plays a role in increasing the risk of gastrointestinal mucosal injury, even bleeding.^[13,14]^

DIE is a rare complication that has occasionally been reported.^[3–12]^ Its endoscopic manifestations include abscission of longitudinally sloughing casts in the middle and/or the lower segment of the esophagus. Some researchers have suggested that dabigatran capsules containing tartaric acid might cause injury to the esophagus after long-term administration. Chest pain, heartburn, odynophagia and dysphagia are the main symptoms. Not drinking enough water, lying down after taking the drug, or decreased salivary secretion increase the chance of contact of dabigatran with the esophageal mucosa, thereby inducing the development of esophagitis. Normally, DIE can be reversed, with good prognosis. Therefore, early diagnosis seems to be important. According to the current consensus on preventing or improving DIE, the patient should take a full glass of water (8 ounces/240 ml) when taking the drug followed by standing for at least 30 minutes. Patients should not crush, chew, or break open the capsules. When serious adverse reactions occur, the patient should stop taking dabigatran immediately and/or replace it with other oral anticoagulant drugs. If necessary, PPIs, such as omeprazole and rebamipide, should be used.^[15]^ However, in our literature review, a 78-year-old woman with DIE who was resistant to omeprazole administration rapidly improved without the need to discontinue dabigatran after being advised to drink a sufficient amount of water and maintain an upright position immediately after drug ingestion.^[9]^ As per reported studies, there is no connection between the dosage of dabigatran and esophagitis. We searched the relevant literature on PubMed (from the databases created to November 2019) that focused on the title “dabigatran” and “esophagitis.” All the patients with esophagitis induced by dabigatran took the medicine according to the instructions at 110 or 150 mg bid. Other risk factors are related to gender (male) and age (old age). Clinical characteristics of DIE case reports on PubMed are summarized in Table 1.^[3–12]^

**Table 1:**
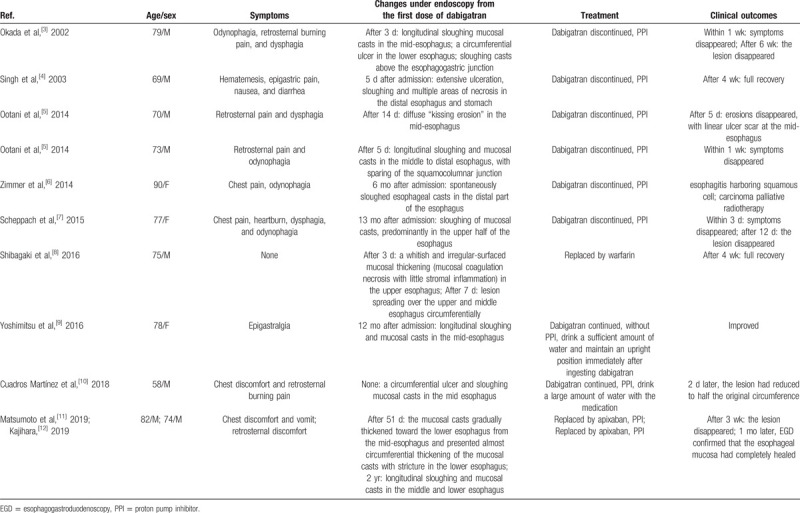
Clinical characteristics of dabigatran-induced esophagitis case report.

In this case, we diagnosed DIE based on the patientʼs history (the patient was on oral dabigatran at 110 mg bid), typical endoscopic manifestation (mucosal congestion and erosion at the esophagus), and pathology (esophageal squamous epithelium with neutrophil infiltration and partial epithelial degeneration). In fact, there is no international consensus on the cause and treatment of esophagitis induced by dabigatran. Unlike most previous cases, our patient developed DIE after taking dabigatran for about 1 year rather than for the first time or only for a short period, indicating that long-term use of dabigatran also results in DIE. So, the indications for this drug should be more carefully considered. In addition, the first EGD examination suggested *H. pylori* infection. The patient's clinical symptoms, endoscopic manifestations, as well as pathological results significantly improved after standard *H. pylori* eradication therapy. However, we do not know whether *H. pylori* infection caused and aggravated DIE. The association between esophagitis and *H. pylori* infection is a complex issue.^[16,17]^ Some researchers argue that *H. pylori* infection is correlated with the pathogenesis of reflux esophagitis.^[18]^ Adachi et al have found that the risk of reflux esophagitis in individuals following eradication of *H. pylori* is lower as compared with those who are never infected. Oikawa et al have suggested that ND1+32656 GG and IL-8-251 T/T alleles may increase the risk of erosive esophagitis, even in an *H. pylori*-infected Japanese population.^[19]^ Yucel has reported that the symptoms of gastroesophageal reflux disease improve after eradication of *H. pylori* in patients with antral gastritis and duodenal ulcers that have hyperacidity.^[20]^ Although our patient had clinical remission, the third EGD still revealed persistent esophageal injury. Therefore, PPIs or other gastric mucosal protective agents are recommended for oral administration synchronously with dabigatran when necessary, even if clinical symptoms are absent.

## 4 Conclusion

We present a case of DIE with *H. pylori* infection in a female patient, who recovered by standard *H. pylori* eradication therapy and PPI without stopping dabigatran. Our case highlights the importance of physicians’ awareness of the clinical and endoscopic characteristics of DIE, and the importance of *H. pylori*-associated tests and eradication if necessary for patients with long-term dabigatran treatment.

## Author contributions

**Conceptualization:** Zhiquan Fu.

**Data curation:** Yi Zhou, Lei Lu.

**Formal analysis:** Yi Zhou.

**Funding acquisition:** Yancheng Dai.

**Investigation:** Yancheng Dai.

**Methodology:** Yancheng Dai.

**Writing – original draft:** Yi Zhou, Yancheng Dai, Zhiquan Fu.

**Writing – review & editing:** Yi Zhou, Yancheng Dai, Zhiquan Fu.
